# In vitro antibacterial effects of photodynamic therapy against Enterococcus faecalis in root canals of deciduous teeth

**DOI:** 10.1186/s12903-022-02523-5

**Published:** 2022-12-01

**Authors:** Meimei Li, WenChee Wong, Huacui Xiong, Ke Chen

**Affiliations:** Stomatological Hospital, Southern Medical University, Guangzhou, China

**Keywords:** Photodynamic therapy, Tooth, Deciduous, Enterococcus faecalis, Root canal irrigants, Confocal laser scanning microscope

## Abstract

**Objective:**

This study aimed at evaluating the in vitro antibacterial efficacy of photodynamic therapy (PDT) on planktonic *E. faecalis* and its biofilm in the root canal of infected deciduous teeth.

**Methods:**

Forty root canals of maxillary deciduous anterior teeth were enlarged up to #35 K-file and inoculated with *E. faecalis* for 21 days. The root canals were randomly assigned into four groups (*n* = 10): The normal saline group (control), 1% NaClO group, PDT group, and the 1% NaClO + PDT group. Paper point samples were obtained at baseline (S1) and after treatment (S2). The colony-forming units (CFU) were counted, and the bacterial growth rate calculated. From each subgroup, 5 samples were randomly selected after treatment and a scanning laser confocal microscope (CLSM) used to determine the distribution of dead / living bacteria on the biofilm surface of each subgroup. A scanning electron microscope (SEM) was used to observe bacterial morphologies in the root canal walls of the remaining 5 samples in each subgroup. The Kruskal–Wallis test and Dunn test with boferroni adjustment were used to analyze the effect of the different treatment techniques on the E. faecalis in root canals.

**Results:**

Compared to the saline group, PDT significantly reduced bacterial counts in the root canal (*p* < 0.05). The CFU counts were lowest (*p* < 0.05) in the 1% NaClO and in 1% NaClO + PDT groups. The rate of bacterial death on the surface of the biofilm in the PDT group was significantly increased after treatment (*p* < 0.05), and the rate of bacterial death was highest in 1%NaClO group and 1%NaClO + PDT group (*p* < 0.05).

**Conclusion:**

PDT has an antibacterial activity against *E. faecalis* in the root canal of deciduous teeth. Its activity against planktonic *E. faecalis* is better than the activity on the intact biofilm. The antibacterial activity of PDT on *E. faecalis* in root canals of deciduous teeth is lower compared to that of 1% NaClO.

## Introduction

Dental carries incidences in deciduous teeth is a public health concern. Due to its rapid progression pulp and periapical disease incidences of deciduous teeth are high [1]. Pulpectomy is a difficult procedure to successfully conduct, and it is particularly challenging to effectively preserve deciduous teeth with pulpitis or periapical periodontitis.The complexity of the root canal system of deciduous teeth makes the irrigation can not disinfect the root canals thoroughly. After treatment, the root canal system of deciduous teeth is prone to reinfections. Due to poor prognosis, the number of deciduous teeth extracted after root canal treatment is high [2]. Children are less likely to cooperate during treatment. The unpleasant treatment experience in young children is the most important factor contributing to behavioral management problems among children. Clinical studies have shown that the success rate of pulpectomy is about 80%, whereas the imaging success rate is lower than the clinical success rate [3, 4]. The rate of success of pulpectomy for the infected teeth is low [4, 5].

Currently, NaClO is the most effective germicidal drug. It has efficient antimicrobial activity and ability to promote pulp dissolution [6]. NaClO is an odorous corrosive liquid with strong cytotoxicity, and this makes children uncooperative during treatment. Deciduous teeth often show apical resorption, When inadvertently injected outside the root tip or soft tissue, NaClO can cause pain, skin ulcers, facial swelling and ecchymosis, choking and asphyxia, and life-threatening in serious cases [6]. In the narrow part of the root canal, the surface tension of NaClO limits diffusion and, therefore, has poor clinical outcomes. The higher the concentration and temperature of NaClO, the higher the antibacterial activity. However, a very high concentration and long exposure time causes serious dentin erosion [7]. To effectively control root canal infection of deciduous teeth in children, a convenient, comfortable and effective root canal disinfection method should be developed. Of these new methods, PDT is one of the most promising [8].

The concept of photodynamic inactivation requires microbial exposure to either exogenous or endogenous photosensitizer(PS) molecules, followed by visible light energy, typically wavelengths in the red/near infrared region that cause the excitation of the PS resulting in the production of singlet oxygen and other reactive oxygen species that react with intracellular components and consequently produce cell inactivation and death[9]. So far, it is not clear which between NaClO and PDT has superior germicidal effects. Several studies on *E. faecalis* infected root canal show that the use of PDT alone can effectively reduce the number of *E. faecalis* bacteria. PDT can effectively disinfect root canal and but cannot replace the traditional chemical irrigation currently. It is therefore a promising auxiliary disinfection method [9]. Other reports indicate that PDT has similar or even stronger germicidal effects as NaClO [10]. Some studies found that the germicidal effects of PDT were not significant. Moreover, administration of PDT after traditional chemical–mechanical cleaning did not improve significant germicidal effects [11].

The advantages of PDT are: it has a non-irritating odor, it is easy to administer and is painless. These features make it ideal as a therapeutic option in children [9]. Differences in the anatomy, histology and chemistry between deciduous teeth and permanent teeth have been well elucidated. The anatomical morphology of the pulp cavity of deciduous teeth is more complex, dentin thickness is smaller, it has a low degree of mineralization and tissue hardness, it has a higher density of dentinal tubules, and the dentin is easy for corrosion. There is a paucity of data on the effects pulpectomy with PDT.

The aim of the study was to determining the in vitro disinfective effects of PDT on the root canal of infected deciduous teeth. The null hypotheses were: (1) there is no difference in CFU counts after the contact with PDT; (2) PDT does not alter the number of viable bacterial cells assessed with confocal laser scanning microscopy (CLSM)and scanning electron microscope (SEM).

## Materials and methods

### Sample collection and preparation

A total of 44 maxillary deciduous anterior teeth obtained from the Department of Pediatric Dentistry, Stomatological Hospital, Southern Medical University were used. The study was approved by the Human Research Ethics Committee of our hospital (No 2019–32).

The inclusion criteria were: i. a complete absence of dental caries or an absence of pulp infection and ii. single-rooted with complete root. To standardize the roots, crowns were removed from the 9 mm roots and the pulps extracted. The working length was established to be 1 mm lower than the apical foramen, so, the root canals were enlarged to #35 K-file (Dentsply-Maillefer, Ballaigues, Switzerland). 1% NaClO (Fujian weizhenyuan medical science and technology Co., Fujian, China) was used as the irrigation solution. To remove the smear layer, the root canals were rinsed in 17% EDTA (Yongan Laboratory, Shandong, China) for 4 min while the removal of residual irrigations was done in 5 ml of normal saline.

An adhesive system (Dentsply, Konstanz, Germany) and a composite resin (Densberg, Milford, USA) were used to seal the apices. The roots were placed in a glass bottle containing distilled water and sterilized in an autoclave (Shanghai Boxun Co., Shanghai, China) at 121 ℃, 1.5 MPA, for 15 min. Two root samples were randomly selected and incubated in brain–heart infusion (BHI) broth (Hukai microorganism Co., Guangzhou, China) for 48 h at 37 °C. Two samples were randomly selected for observation under SEM(Hitachi, Japan) to make sure that the smear layer of the root canal wall was removed.

The root canals were infected with a standard strain of *E. faecalis* (ATCC29212). *E. faecalis* was grown in BHI broth at 37 °C overnight. At a wavelength of 600 nm, its concentration was spectrophotometrically adjusted to an absorbance of 0.1 (about 1 × 10^8^ cells/ml).

The root canal samples were placed on the silicone rubber base in a 24-well plate. A 2 ml aliquot of the culture of *E. faecalis* was inoculated into the root canal of each sample. To promote growth, the *E. faecalis* cultures were maintained for 21 days. After every 48 h, BHI was replaced.

### Disinfection of the root canals

Forty samples were randomly assigned into 4 groups of 10 each.

Group 1(Normal saline group): The root canals were irrigated with 5 ml of saline for 1 min. To rinse the root canals, a 27-G lateral perforated needle was introduced 2 mm short of the working length, pull up and down slightly.

Group 2(1% NaClO group): 5 ml of 1% NaClO was used to irrigate the root canal for 2 min after which they were irrigated with 5 ml of saline for 1 min.

Group 3(PDT group): 0.1 mg/ml methylene blue (Zhengzhou Jiatai Biotechnology Co., Ltd., Zhengzhou, China) was injected into the root canal for 1 min. Next, following the manufacturer’s instruction,diode laser (Zhengzhou Jiatai Biotechnology Co, Ltd, Zhengzhou, China), with 13.2 J/cm^2^, 200mW and red continuous emission (660 ± 10 nm wavelength), with an intracanal fiber (diameter 0.6 mm) attached, was used to irradiate the apical third for 1 min and this process was conducted twice. Subsequently, 5 ml saline was used to irrigate the root canal for 1 min.

Group 4(1% NaClO + PDT group): Refer to group 2,the PDT procedure was performed and the diode laser irradiate for 1 min twice.Then 5 ml of 1% sodium hypochlorite was used to rinse the root canals for 2 min after-which they were rinsed with 5 ml of normal saline for 1 min.

### Microbiological analysis

The samples collected immediately after contamination with *E. faecalis* and before the decontamination process were referred to as the initial sample (S1), while the samples collected after the decontamination process were referred to as the final sample (S2). A sterile size 35 paper point(Daya Ding Medical Devices Co., Ltd., Beijing, China)was placed in the root canal for 1 min and transferred to a sterile Eppendorf tube containing 1 ml saline. The suspension in the Eppendorf tube was vortexed for 60 s. Tenfold serial dilutions of the suspension was done in saline. 0.1 ml aliquots were spread on BHI agar plates and incubated at 37 °C for 48 h. Colony counts were done after incubation. The operator who is conducting the experiment and the examiners when assessing the results must be provided are different people.

### Sample preparation for confocal laser scanning microscopy

Five root canals of deciduous teeth were randomly selected from each group and split using a slow turbine. Each sample was treated with LIVE/DEADTM BacLight Bacterial Viability Kit-L7012 (Invitrogen, Oregon, USA) staining solution for 15 min. Staining was done in the dark at room temperature. Residual fluorescent dyes were removed by rinsing in PBS for 1 min. Samples on the confocal plate were observed by CLSM (LSM 880 with Airyscan, ZEISS,Germany). From each sample, three fields of view were randomly selected and the CLSM image of the surface layer of the biofilm (1024 × 1024 pixels) was observed under a × 20 lens. The green channel represented living bacterial cells while the red channel represented dead bacterial cells. The fluorescence intensity of the image is analyzed using Image J software, and the ratio of red fluorescence to red fluorescence + green fluorescence, that is, the ratio of dead bacteria to total bacteria, was calculated.

### SEM preparation and analysis

After treatment, the root canals and the remaining 5 samples in each group were detected by SEM. Samples were stored in glutaraldehyde solution at 4 ℃ for 24 h and were then detected by a scanning electron microscope after gradient dehydration, critical point drying and gold spraying. Magnifications of × 1000, × 5000 and × 20,000 were used focus the residual biofilm on the surface of the root canal.

### Data analysis

The statistical software SPSS 25 was used in data analysis. Normal distribution and data homogeneity were tested. Since the data were not normally distributed, the Kruskal–Wallis test and Dunn test with boferroni adjustment were used to analyze the effect of the different treatment techniques on the *E. faecalis* in root canals. Values with *p* < 0.05 were considered significantly.

## Results

Table 1 shows the median of *E. faecalis* colony count (× 10^2^ CFU/ml), the 25^th^ and 75^th^ percentiles, and the percentage reductions of S1-S2 bacteria. Before treatment (S1), the statistical differences in *E. faecalis* colony counts in the four groups were not significant (*p* > 0.05). After treatment (S2), compared to the saline group, the colony counts in the root canals of the three groups were lower after treatment.There was a significant decrease (99.33%) in colony counts in the PDT group. Colony counts in the 1% NaClO and 1% NaClO + PDT groups were lower compared to the PDT group. The statistical differences in colony counts between the 1% NaClO group and the 1% NaClO + PDT group were not significant.

**Table 1:**
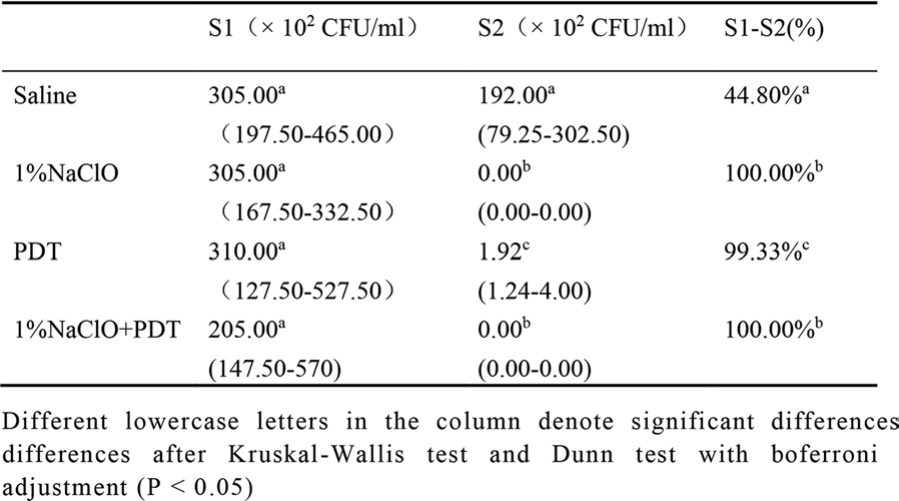
*E. faecalis* colony counts of baseline(S1) and after treatment(S2) after decontamination process

**Fig. 1:**
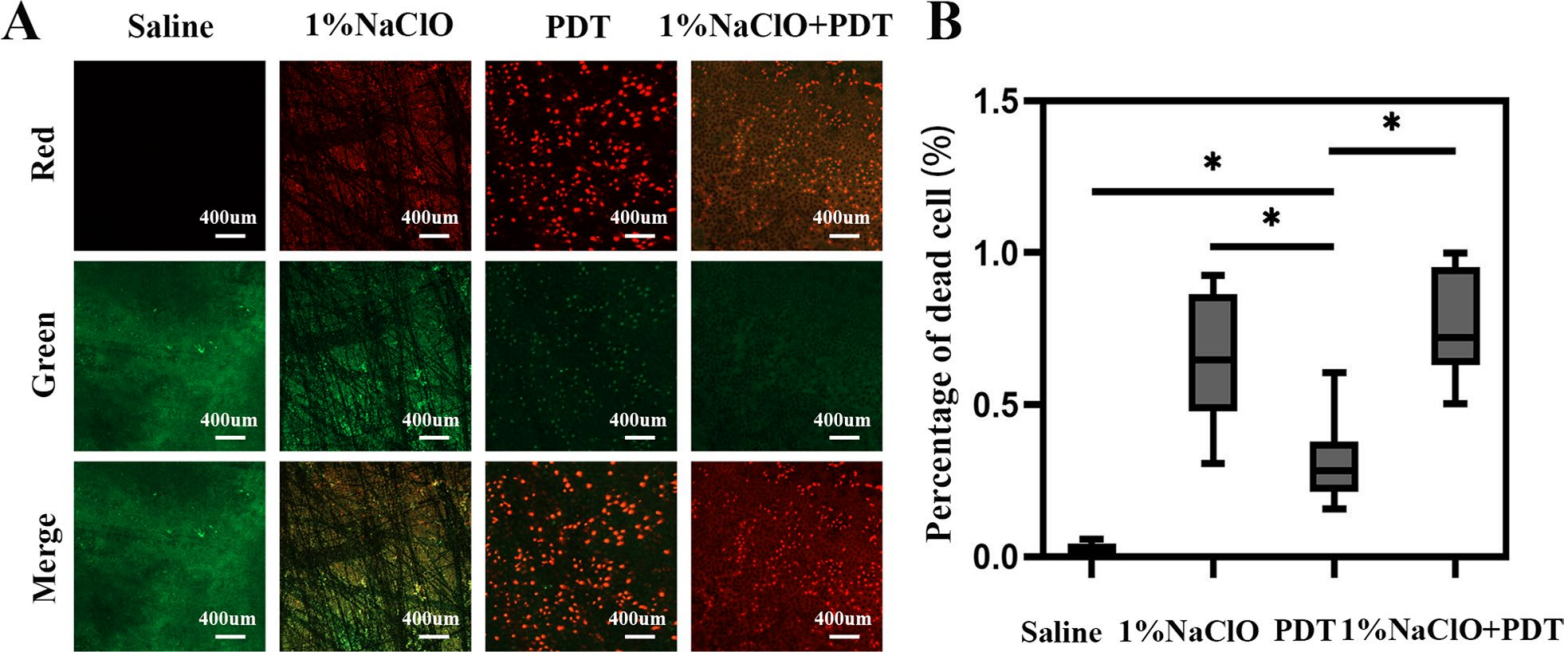
(A) CLSM pictures of biofilm on the root canal surface after decontamination process. Green fluorescene indicates live cells, and red fluorescene indicate dead cells. Images are representative examples of four groups. (B) The rate of bacterial death of biofilm on the root canal surface after decontamination process

Figure 1 show the representative CLSM images of biofilm and proportion of dead bacteria of biofilm on the surface of root canals after treatment. It is shown that the proportion of dead bacteria on the biofilm surface of root canals in the experimental groups were higher than in the saline group. In addition, the proportions of dead bacteria in the 1% NaClO group and 1% NaClO + PDT group were higher compared to the PDT group. There was no significant difference in the proportions of dead bacteria between the 1% NaClO + PDT group and the 1% NaClO group.

**Fig. 2:**
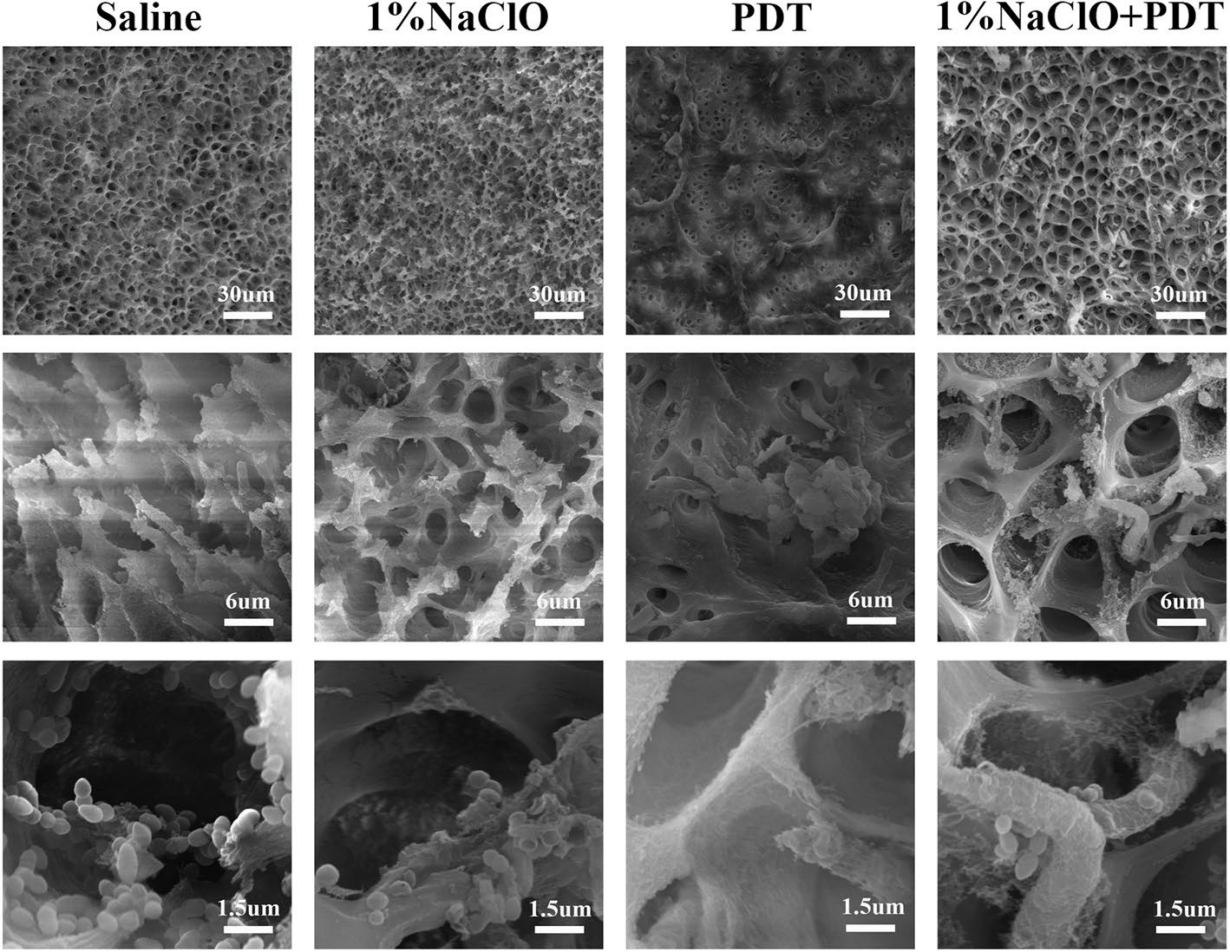
The SEM pictures of biofilm on the surface of root canals after decontamination process

Figure 2 shows the SEM images of biofilms on the surface of root canals after treatments. The residual biofilm was detected on the root canal surface of the four groups. In the normal saline group, a significant bacterial count was clustered in the root canal wall and dentinal tubules and connected to form a biofilm. In the 1% NaClO + PDT and 1% NaClO groups, the bacterial biofilms were seriously damaged as most of the bacteria were degraded and deformed. In the PDT group, the biofilm was destroyed but, a significant number of bacteria remained intact.

## Discussion

Effective root canal disinfection improves clinical outcomes in pulpectomy [12]. E. faecalis is one of the most common microorganisms in infecting the root canal system of deciduous teeth, and highly associated with pulp necrosis, periapical shadow and pain [13]. E. faecalis can survive and reproduce in the environment of strong alkali and nutrient deficiency, and can form a single biofilm. Therefore, it has strong resistance to root canal drugs such as calcium hydroxide and has strong toxicity [14]. Routine root canal preparation and irrigation are difficult to completely kill or remove it [15]. Therefore, one of the key factors for successful pulpectomy is the complete elimination of E. faecalis in the root canal. Therefore, for a long time, scholars have often used E. faecalis to infect isolated teeth and establish root canal infection models in vitro to evaluate the efficacy of root canal disinfection drugs [10, 11]. In this study, an in vitro model of root canal of deciduous teeth infecting E. faecalis was established,to study the bactericidal effect of PDT.In previous studies,PDT was applied in vivo to disinfect root canal of deciduous teeth using the bacterial culture method of sterile paper tip sampling [16]. This method can detect the number of bacteria planktonic but the sensitivity is low [17]. A combination of CLSM with fluorescence staining clearly shows the distribution of dead bacteria in the biofilm and calculates their proportions. This technique has a high sensitivity and can be easily duplicated. This method is best used when observing and evaluating bacterial activities in root canals [18]. SEM can directly and clearly observe the distribution of E. faecalis and the morphology of bacteria on the sample surface [19]. In this study, bacterial culture, CLSM and SEM were used to determine the antibacterial activities of PDT on E. faecalis in root canals of deciduous teeth.

To achieve high efficacies, NaClO is usually used in the 0.005–5.25% concentration ranges [7]. The organic matter content and water content of deciduous teeth have been found to be higher compared to permanent teeth. In addition, deciduous dentin responses to chemical washing agents are stronger compared to permanent teeth [20]. A high concentration of NaClO causes dentin corrosion and increases the risk of periapical spillover due to physiological root resorption of deciduous teeth. Comparatively, the antibacterial effect of high concentration of NaClO and that of heavy use of 1%NaClO flushing is not significantly different [21]. Therefore, we used 1% NaClO as the positive control.

The effectiveness of PDT is mostly related to three main aspects: (i) PS capability of interacting with the bacterial membrane; (ii) PS ability of penetration and action inside the cell; and (iii) reactive singlet oxygen formation around the bacterial cell by illumination of the PS [9]. MB is a cationic PS with amphiphilic (hydrophobic and hydrophilic) properties, has better permeability to biofilm. It was found that PDT with MB was able to damage the biofilm structure to a greater extent [22]. The advantages of laser technique are monochromaticity and high efficiency. The semiconductor laser is cheap, convenient and reliable. This makes it highly applicable in most PDT studies [9]. In this study, MB was used as a PS while the low intensity semiconductor laser was used as light source in the determination of the effects of MB-mediated PDT on root canal disinfection of deciduous teeth.

We showed that the number of S1 bacteria in the four groups was very high and the statistical difference between the four groups was not significant. This confirmed bacterial colonization before treatment. PDT significantly reduced colony counts in the root canal by up to 99.33%. Colony counts in the 1% NaClO group was lower compared to the PDT group. When combined with effective flushing, 1% NaClO exhibited a good antibacterial effect on planktonic *E. faecalis*. The reduction rate of bacteria in the 1% NaClO group was 100%. However, due to experimental limitations proof that PDT in combination with 1% NaClO can improve antibacterial effects is elusive.

To detect the antibacterial efficacy of PDT on mature biomass membrane in root canals, the CLSM detection method was used. After treatment with PDT or 1% NaClO, the death rate of bacteria on the biofilm surface increased significantly. This suggested that both PDT and 1% NaClO had an effective bactericidal effect on *E. faecalis* biofilm formation. The proportion of dead bacteria in the PDT group is less compared to the 1% NaClO group. This shows that the antibacterial effects of PDT are weak compared to 1%NaClO. But there was no significant difference between 1%NaClO group and PDT + 1% NaClO group. It is likely that in this experiment, to reduce the staining of MB on teeth [23]. We rinsed the teeth with NaClO after PDT treatment, and hence NaClO was dissolved and washed away the biofilm bacteria killed by PDT. Therefore, the germicidal efficacy of PDT was not significant. The volume of residual biofilm and distribution of dead bacteria at different depths need to be detected next step. Nevertheless, our sample size was small, and the chosen pictures could be biased. A larger sample size is needed for further study.

In the biofilm, bacteria are wrapped by outer bacterial macromolecules that make them difficult to be targeted by the immune system or drugs [24]. In clinical practice, mechanical preparation is indispensable. Root canal disinfection and irrigation is only one of the approaches used to remove infections. PDT must be carried out after root canal preparation, should not be conducted alone. Therefore, during clinical treatment, most bacteria in the non-infected root canals and mechanically prepared bacteria exist in a planktonic state, and intact biofilms mainly exist in areas where physical cutting leakage is limited by irregular morphology [25]. When the biofilm structure is intact and not partially destroyed by irrigation agents, or root canal preparation machinery, the antibacterial effects of PDT are poor [26].

PDT possess antibacterial effects. Our results are consistent with those reported in other studies [10, 15]. It has been shown that PDT has similar antibacterial effects as NaClO [14]. This deviation from our findings could be due to differences in experimental methods, PS and light sources. In addition, PDT is more effective against Gram-positive bacteria than Gram-negative bacteria[27]. Differences in the type, concentration and formulation of the PS will affect its penetration power into bacteria and ability to form active singlet oxygen after light irradiation, thus affecting the disinfection efficacy of PDT [28]. The sources of light and energy dose affect active singlet oxygen formation after light irradiation, thereby affecting the disinfection effect of PDT on root canal [29]. There are no established guidelines for the formulation of a PS, the energy dose, light source and the optimal exposure time. More studies are needed to standardize and optimize PDT parameters in order to establish a safe, repeatable and effective PDT regimen for clinical applications [9]. In addition, PDT was reported to enhance the apical tissue healing. PDT with low photosensitizer concentration and low doses of laser energy density may improve osteogenic differentiation of apical papilla stem cells [30].Therefor, more in vivo studies are needed to verify these findings.

## Conclusion

PDT has an antibacterial activity against *E. faecalis* in the root canal of deciduous teeth. Its activity against planktonic *E. faecalis* is better than the activity on the intact biofilm. The antibacterial activity of PDT on *E. faecalis* in root canals of deciduous teeth is lower compared to that of 1% NaClO.

## Data Availability

The dataset used and/or analyzed during the current study is available from the corresponding author on reasonable request.
